# Concerns, perceived impact, and preparedness of oral healthcare workers in their working environment during COVID‐19 pandemic

**DOI:** 10.1002/1348-9585.12168

**Published:** 2020-09-20

**Authors:** Farooq Ahmad Chaudhary, Basaruddin Ahmad, Paras Ahmad, Muhammad Danial Khalid, Danial Qasim Butt, Soban Qadir Khan

**Affiliations:** ^1^ Dental College HITEC Institute of Medical Sciences Taxila Pakistan; ^2^ School of Dental Sciences Universiti Sains Malaysia Kubang Kerian Malaysia; ^3^ AO Research Institute Davos AO Foundation Davos Platz Switzerland; ^4^ College of Dentistry Iman Abdulrahman Bin Faisal University Dammam Saudi Arabia

**Keywords:** concerns, COVID‐19, impact, oral healthcare worker, preparedness

## Abstract

**Objective:**

The aim of the study was to evaluate the oral healthcare workers' concerns, perceived impact, and preparedness in COVID‐19 pandemic.

**Methods:**

This cross‐sectional study was carried out at 10 different dental hospitals in Pakistan from March to June 2020. A 35 items valid and reliable questionnaire was used to assess the concerns, perceived impact, and preparedness of oral healthcare workers (OHCW) in COVID‐19 pandemic. Chi‐squared test and logistic regression were used for analysis.

**Results:**

A total of 583 OHCW participated in this study. The odds of having the awareness about the risk of exposure and fear of getting infected, were greater in the clinical than non‐clinical OHCW (OR: 52.6; OR: 15.9). For social network concerns, the clinical OHCW were more likely to be concerned about their colleagues (OR: 6.0). The clinical OHCW have greater odds of worrying about telling the family/friends about the risk exposed to (OR: 2.55), being avoided because of the job (OR: 3.20) and more likely to be feeling stressed (OR: 4.31). Less than 50% of the participants felt that their institutions are well prepared and only 12.6% had attended an infection control training session. Most participants practiced self‐preparation such as buying masks and disinfection (94.3%, 98.3%).

**Conclusion:**

The majority of OHCW felt concerned about their risk of exposure to infection and falling ill from exposure and infecting friends/family. There is a need for training of infection control and PPE and minimizing fear and psychological impact on OHCW should be the priority in any preparedness and planning for combating COVID‐19.

## 1 INTRODUCTION

The World Health Organization (WHO) had declared the novel coronavirus (COVID‐19) a pandemic on the 11th of March 2020.^1^ At the time of writing this article, the virus has spread over 213 countries and affecting 8 million people worldwide, with a case fatality rate of 2.3% in China and 1.6% outside China.^2^ In Pakistan, the first case of Covid‐19 was confirmed on 26 February 2020, when a student in Karachi was tested positive upon returning from Iran.^3^ As of 15 June 2020, there are about 144 500 confirmed cases with 53 700 recoveries and 2730 deaths in the country.^2^ The ratio of positive cases to tests also increased, hovering around 20%‐25% in the first few weeks of June.^4^

The strain that the pandemic has placed on the healthcare facilities across the world is unprecedented and extraordinary measures are adopted to meet the challenges.^5^ Hospitals have made the use of masks, gloves, and gowns mandatory for all staff who attend to patients or interact with each other; quarantined thousands of people and recommended that healthcare workers (HCW) not to have any contact outside work duties.^3^, ^4^ There are hospitals with a persistent shortage of ICU beds, ventilators, personal protective equipment (PPE), and other medical equipment. These increase the risk of exposure among the HCW when attending to COVID‐19 infected patients and generates fear and stress of contracting the virus.^6^, ^7^

According to the International Council of Nurses (ICN) at least 90 000 healthcare workers worldwide are believed to have been infected with COVID‐19, and possibly twice that, amid reports of continuing shortages of protective equipment.^8^ In Pakistan, more than 500 healthcare workers are believed to have been infected with COVID‐19 by now which leads to a surge in complaints about the lack of personal protection equipment (PPE) in hospitals.^9^ It is required to deal with the coronavirus, and that without protection doctors have become infected and in turn, were infecting other patients.

Previous researches on COVID‐19 infection among the HCW had focussed on the concerns, impact, and preparedness of those working in general medicine and surgery,^10^, ^11^ and to our knowledge, there is no study on oral healthcare workers (OHCW) has been reported. The OHCW faces similar challenges to the HCW, if not greater, because of the high risk of cross‐infection during clinical practice.^12^ Clinical OHCW, particularly the dentist, are exposed to fluid and aerosols, particularly from the oral cavity during a common dental procedure such as dental scaling, restoration, and extraction.^12^, ^13^ Because an individual with COVID‐19 could be asymptomatic for several days without knowing it, they pose a risk to the OHCW when seeking dental treatment. Therefore, this study was aimed to evaluate/contrast the clinical and non‐clinical oral healthcare workers' concerns, perceived impact, and preparedness in a COVID‐19 pandemic in dental hospitals of Pakistan.

## 2 METHODS

This cross‐sectional study on OHCW was carried out at 10 different dental hospitals in three provinces (Punjab, Sindh, and KPK) and the capital administration territory of Pakistan from March to June 2020. Ethical approval for the study protocol was obtained from the Dental College, HITEC Institute of Medical Sciences (Reference no. F.23‐11C/2020/ERB/HITEC‐IMS). All the OHCW at the dental hospitals were identified by the administrator of the respective institutions and included in the study. They were provided with an explanatory letter that explains the purpose of the survey, consent form, and a self‐administered questionnaire. Participation in the study was voluntary.

The OHCW were categorized into two groups; the clinical (dentist and dental assistant/hygienist) and non‐clinical groups (dental laboratory technician, attendant/cleaners, managerial/clerical services) based on the interaction with the patients.

The questionnaire collected socio‐demographic and COVID‐19 related information. The socio‐demographic information included the age (categorized as 20‐29, 30‐39, 40‐49, 50‐60), sex (male, female), marital status (single, married, divorced, widowed), place of work (government/public sector, private sector), and place of living (family/friends, alone). The concerns, perceived impact, and preparedness of OHCW in the COVID‐19 pandemic were assessed using a valid and reliable questionnaire modified and adopted for the COVID‐19 pandemic from Wong et al (2008). The questionnaire consisted of 35 questions in four sections. The first two sections have eight questions each and assess the work and social concerns of the OHCW during the pandemic. The third section has nine questions that assess the perceived impact of a pandemic and the last section with 10 questions is related to the preparedness to face the pandemic. The response for each question ranges from 1 (strongly disagree) to 6 (strongly agree) on a 6‐point Likert scale and was dichotomized as “agree” (strongly agree, agree, and probably agree) and “disagree” (strongly disagree, disagree, and probably disagree). The adopted translated Urdu questionnaire was administered on 25 dentists for assessing its reliability and validity and shown to have a satisfactory internal consistency with Cronbach's α 0.81.

### 2.1 Statistical analysis

Summary statistics were obtained for all variables. Chi‐squared tests and logistic regression were used to compare parameters between the clinical and non‐clinical staff. All analysis was performed at a 5% significance level and carried out in IBM SPSS software version 25.0 (SPSS Institute, Chicago, IL, USA).

## 3 RESULTS

A total of 583 OHCW from 10 different dental hospitals in three provinces and the administration capital of Pakistan had participated in the study. The majority were below the age of 40 years (62.8%) and there were significantly more female clinical than non‐clinical staff (*P* < .001) (Table 1). The distribution of participants by marital status, place of work, and living arrangement was similar in the two groups.

**Table 1:** Characteristics of oral healthcare workers

	Clinical staff, N = 392 (67.2), N (%)	Non‐clinical staff, N = 191 (32.8), N (%)	*P*‐value
Age			
20‐29	124 (31.6)	51 (26.7)	.5
30‐39	120 (30.6)	69 (36.1)	
40‐49	98 (25.0)	46 (24.0)	
50‐60	50 (12.7)	25 (13.0)	
Gender			
Male	177 (45.1)	148 (77.4)	.001
Female	215 (54.8)	43 (22.5)	
Marital status			
Single	77 (19.6)	45 (23.5)	.7
Married	301 (76.7)	141 (73.8)	
Divorced	12 (3.0)	4 (2.0)	
Widowed	2 (0.5)	1 (0.5)	
Place of work			
Government/public‐Sec.	205 (52.2)	106 (55.4)	.3
Private sector	187 (47.7)	85 (44.5)	
Staying with			
Family/friend	344 (87.7)	167 (87.4)	.9
Alone	48 (12.2)	24 (12.5)	

In general, most of the participants were aware and concerned about the COVID‐19 pandemic and the risk to their family. The odds of having the awareness about the risk of exposure to COVID‐19 and fear of getting infected, were greater in the clinical than non‐clinical OHCW (OR: 52.6; OR: 15.9, respectively) (Table 2). The clinical OHCW are also more likely to feel that the risk of exposure is not acceptable (OR: 5.3) and accept it as part of the job (OR: 7.9) compared to the counterparts. However, they are less likely to believe that the employers will look after their need in case they are infected than the non‐clinical workers (OR: 0.08). For social network concerns, the clinical OHCW were more likely to be concerned about their colleagues compared to non‐clinical counterparts (OR: 6.0) (Table 3).

**Table 2:** Work‐related concerns regarding a COVID‐19 Pandemic

Concerns (agree)	Clinical, N (%)	Non‐clinical, N (%)	Total, N (%)	Unadjusted OR	*P*‐value	Adjusted OR^a^	*P*‐value
Work‐related concerns							
The job risks an exposure to COVID‐19	386 (98.5)	105 (55.0)	491 (84.2)	64.4 (25.8‐161.1)	.001	52.6 (22.4‐123.9)	.001
Fear of getting infected by COVID‐19	370 (94.4)	98 (51.3)	461 (80.3)	17.5 (9.81‐31.4)	.001	15.9 (9.5‐26.7)	.001
Should not care for COVID‐19 patients	388 (99.0)	191 (100)	579 (99.3)	‐	‐	‐	‐
The risk is not acceptable	388 (99.0)	181 (94.8)	569 (97.6)	5.0 (1.41‐18.3)	.013	5.3 (1.65‐17.3)	.005
The risk is part of the job	385 (98.2)	167 (87.4)	552 (94.7)	6.9 (2.74‐17.4)	.001	7.9 (3.34‐18.7)	.001
Consider looking another job because of risk	63 (16.1)	21 (11.0)	84 (14.4)	1.10 (0.61‐1.95)	.7	1.55 (0.91‐2.62)	.1
Acceptable if colleagues resign because of their fear	373 (95.2)	178 (93.2)	551 (94.5)	0.20 (0.75‐3.66)	.2	1.43 (0.69‐2.96)	.3
Healthcare employers would look after my needs if I fall ill with COVID‐19	225 (57.4)	180 (94.2)	405 (69.5)	0.083 (0.43‐0.16)	.001	0.08 (0.43‐0.15)	.001

^a^ Adjusted for age, gender, Marital status, Place of work, Staying with.

**Table 3:** Non‐work‐related concerns regarding a COVID‐19 Pandemic

Concerns (Agree)	Clinical, N (%)	Non‐clinical, N (%)	Total, N (%)	Unadjusted OR	*P*‐value	Adjusted OR^a^	*P*‐value
Non‐work concerns							
People close to me would be at high risk of getting COVID‐19 because of my job	386 (98.5)	185 (96.9)	571 (97.6)	1.45 (0.40‐5.17)	.6	2.08 (0.66‐6.55)	.2
I would be concerned for my:							
Spouse/partner	305 (77.8)	142 (74.3)	447 (76.7)	0.61 (0.08‐4.51)	.6	1.21 (0.80‐1.81)	.4
Parents	235 (59.9)	104 (54.5)	339 (58.1)	1.03 (0.67‐1.57)	.9	1.25 (0.88‐1.77)	.2
Children	235 (59.9)	126 (66.0)	361 (61.9)	0.64 (0.33‐1.22)	.2	0.77 (0.53‐1.10)	.2
Close friends	180 (45.9)	94 (49.2)	274 (47.6)	0.85 (0.54‐1.33)	.5	0.87 (0.63‐1.23)	.5
Work colleagues	369 (94.1)	139 (72.8)	508 (87.1)	9.06 (4.67‐17.5)	.001	6.0 (3.53‐10.1)	.001
People close to me would be worried for my health	359 (91.6)	177 (92.7)	536 (91.9)	0.83 (0.39‐1.73)	.6	0.86 (0.44‐1.64)	.7
People close to me would be worried as they may get infected by me	336 (85.9)	170 (89.0)	506 (86.9)	0.60 (0.32‐1.11)	.1	0.75 (0.44‐1.28)	.3

^a^ Adjusted for age, gender, marital status, place of work, staying with.

Most participants worried about the impact COVID‐19 pandemic on personal, social, and work‐life (Table 4). The clinical OHCW, compared to the non‐clinical counterparts, have greater odds of worrying about telling the family/friends about the risk they are exposed to (OR: 2.55) and being avoided because of the job (OR: 3.20), and avoiding informing others about the nature of the job (OR: 3.19). Also, they are more likely to be concerned about the lack of staff who handles the increased demand, feeling stressed at work (OR: 4.31), and have increased workload (OR: 2.5), and performing work outside the job description (OR: 2.19).

**Table 4:** Perceived impact on personal life and work

Perceived impact (agree)	Clinical, N (%)	Non‐clinical, N (%)	Total, N (%)	Unadjusted OR	*P*‐value	Adjusted OR^a^	*P*‐value
I would be afraid of telling my family/friends about the risk I am exposed	336 (85.7)	134 (70.2)	470 (80.6)	2.99 (1.83‐4.88)	.001	2.55 (1.67‐3.88)	.001
People would avoid me because of my job	351 (89.5)	139 (72.8)	490 (84.0)	4.50 (2.54‐7.97)	.001	3.20 (2.03‐5.04)	.001
People would avoid my family members because of my job	336 (85.7)	129 (67.5)	465 (79.8)	4.26 (2.53‐7.16)	.001	2.88 (1.90‐4.36)	.001
I would avoid telling other people about the nature of my job	347 (88.5)	135 (70.7)	482 (82.7)	5.29 (2.98‐9.38)	.001	3.19 (2.06‐4.96)	.001
There would be inadequate staff at my workplace to handle the increased demand	381 (97.4)	117 (61.3)	498 (85.6)	41.5 (18.1‐95.2)	.001	24.0 (12.0‐48.1)	.001
There would be more conflict amongst colleagues at work	230 (58.7)	107 (56.0)	337 (57.8)	1.03 (0.65‐1.62)	.8	1.11 (0.78‐1.58)	.5
I would feel more stressed at work	356 (90.8)	133 (69.6)	489 (83.9)	6.85 (3.84‐12.2)	.001	4.31 (2.71‐6.83)	.001
I would have an increase in workload	338 (86.2)	137 (71.7)	475 (81.5)	3.13 (1.88‐5.23)	.001	2.46 (1.61‐3.77)	.001
I would have to do work not normally done by me	323 (82.4)	130 (68.1)	453 (77.7)	2.64 (1.64‐4.24)	.001	2.19 (1.47‐3.27)	.001

^a^ Adjusted for age, gender, marital status, place of work, staying with.

Regarding readiness to face the pandemic, less than 50% of the participants felt that the institutions they are working for are well prepared (Table 5). About half of the participants knew the existence of an infection control committee in their institution and received training for infection control. Fewer knew about a preparation plan or received information about it from the hospital. Even fewer had prepared for the pandemic with greater odds in the clinical compared to the non‐clinical OHCW. Very few had attended an infection control training session in the past 6 months (12.6%), and much fewer in the non‐clinical group. Most participants practiced self‐preparation such as buying masks and disinfection (94.3%, 98.3%). The clinical group is more likely to receive training on the use of personal protective equipment than non‐clinical OHCW (OR: 7.51).

**Table 5:** Preparedness for a COVID‐19 Pandemic

Statement (agree)	Clinical, N (%)	Non‐clinical, N (%)	Total, N (%)	Unadjusted OR	*P*‐value	Adjusted OR^a^	*P*‐value
There is an infection control committee in the hospital	198 (50.5)	101 (52.9)	299 (51.3)	0.82 (0.56‐1.19)	.2	0.90 (0.64‐1.28)	.5
I have received training for infection control at my hospital	203 (51.8)	97 (50.8)	300 (51.5)	0.98 (0.67‐1.43)	.9	1.04 (0.73‐1.47)	.8
My clinic has a preparedness plan for COVID‐19 outbreak	161 (41.1)	79 (41.4)	240 (42.2)	0.91 (0.62‐1.33)	.6	0.98 (0.69‐1.40)	.9
My hospital has informed me of the COVID‐19 outbreak preparedness plan	162 (41.3)	79 (41.4)	241 (42.3)	0.92 (0.63‐1.34)	.6	0.99 (0.70‐1.41)	.9
I am personally prepared for a Covid‐19 outbreak	121 (30.9)	75 (39.3)	196 (33.6)	0.68 (0.44‐1.05)	.08	0.69 (0.48‐0.99)	.04
In the past 6 months							
I have attended infection control training sessions	92 (23.5)	5 (2.6)	97 (12.6)	18.0 (6.85‐47.2)	.001	11.4 (4.55‐28.5)	.001
Bought disinfection	386 (98.5)	164 (85.9)	550 (94.3)	13.2 (4.89‐35.5)	.001	10.5 (4.29‐26.1)	.001
Bought masks	391 (99.7)	182 (95.3)	573 (98.3)	20.4 (2.09‐199.0)	.009	19.3 (2.43‐153.7)	.005
Received adequate personal protective equipment training	172 (43.9)	18 (9.4)	190 (42.6)	13.3 (7.32‐24.3)	.001	7.51 (4.44‐12.7)	.001
Have someone to turn to if unsure of use of personal protective equipment	227 (57.9)	96 (50.3)	323 (55.4)	1.55(1.05‐2.29)	.025	1.36 (0.96‐1.92)	.082

^a^ Adjusted for age, gender, marital status, place of work, staying with.

## 4 DISCUSSION

In this study, we assessed the concerns, perceived impact, and preparedness of OHCW who worked at dental hospitals in Pakistan during COVID‐19 pandemic and contrasted between the clinical and non‐clinical staff. The majority of OHCW concerned with the risk of getting infected with COVID‐19 and the job puts them at greater risk of exposure. The findings are consistent with another study on healthcare workers in Singapore during the SARS outbreak.^10^

In this study the majority of OHCW in both clinical and non‐clinical groups felt that they should not look after COVID‐19 patients and risking themselves to the exposure is not acceptable. However, they accepted that the risk of contracting COVID‐19 is part of a job. The findings are in contrast to other studies on HCWs where only a small proportion of participants refuse to look after infectious patients and the majority didn't accept the risk of infection as part of their jobs.^14^, ^15^ The plausible reasons might be related to a higher perception of personal danger, heightened by intense media coverage of this COVID‐19 pandemic and its catastrophic consequences and effects like shortage in Personal protective equipment (PPE) in hospitals, inadequate and insufficient testing and medical supplies, limited treatment options, extended workloads, and other emerging concerns.^16^

Only a small proportion of the participants in this study had considered looking for another job because of a pandemic. The response is consistent with reports from Singapore, and USA in that the majority of HCW are willing to continue working amidst SARS and Avian influenza outbreak respectively.^10^, ^17^ However, other studies showed that a large proportion of HCW in Taiwan, Hong Kong, and the United Kingdom (43%‐77%) was unwilling to work during an infection outbreak, considered looking for another job or quitting their job.^18^, ^19^, ^20^ It may seem unethical to deny treatment by an individual with an infectious disease but the HCW is also concern about their own health and the impact it has on the life and quality of life of their family.^21^ A similar is observed in this study, where the OHCW and their family are worried about the risk of COVID‐19 transmission from the job. This concern could potentially influence the level of commitment of OHCW during a pandemic and should be addressed in any preparedness plan.

The majority of participants are concerned that people will avoid them and their family members because of their occupation. This is also observed during the SARS outbreak and there have several media reports about the discrimination against HCW in uniform and their family members.^22^, ^23^ In the current pandemic, many governments have effectively addressed the prejudice by highlighting the sacrifices, commitment, and dedication of HCW in the media.^24^, ^25^ These have changed public opinion to support the efforts by the HCW to serve the people and country. In Pakistan, law enforcement in several cities has been presented the Guard of Honour as a mark of respect for their services and commitment as a front liner in the fight against COVID‐19.^26^ To show similar support, on 27th March 2020, the celebrities and citizens across Pakistan raised white flags from their balconies and rooftops to express love and gratitude for the doctors and paramedics.^27^ The Government also announced a 1‐month honorarium for the healthcare workers. However, the findings from this study suggest the perception and fear of discrimination against HCW including the OHCW still persist.

Only half of the OHCW in the study received training on infection control and a smaller proportion (42.6%) were trained to use the personal protective equipment. That is lower compared to the HCW in Singapore (88%) who were involved in the infection‐control activities during the SARS outbreak, who had training for infection‐control, outbreak preparedness plan, and effective use of PPE.^10^ The OHCW has a high risk of contracting this infection, thus requires adequate training not only as a prevention but also as a reassurance to help them feel that they are better prepared and maintain high morale while on the job. Many countries including Pakistan have developed plans and guidelines for dental care services during COVID‐19 including safety and training protocols which should be a part of dental hospital infection control and PPE training sessions.^28^, ^29^ There are a few limitations to our study. Recall, framing, and rating bias are some of the limitations of a cross‐sectional survey with self‐administered questionnaires. For example, infection control is commonly taught and briefed to all staffs working in clinical environments, including the attendants, cleaners, and laboratory staffs, because of the risk involved when dealing with human related specimen or derivatives such as dental impression; but because they are not specific to the current pandemic, some may not recall the training. Keeping in mind the rapidly changing situation of the COVID‐19 pandemic, it may be argued that the concerns, perceived impact, and preparedness of OHCW may alter with time as more research on prevention and possible treatment of COVID‐19 will emerge. The key strength of this study was its originality as it is the first study that explored the concerns, impacts, and preparedness of OHCW in the COVID‐19 pandemic. This study helps to pave the way for further COVID‐19 related studies on OHCW.

## 5 CONCLUSION

This study showed that the majority of OHCW are concerned about the risk of COVID‐19 infection, falling ill from exposure and infecting friends/family due to their occupation. Other concerns were related to lack of preparation and few infection control training sessions conducted by their institutions. These concerns could potentially affect the OHCW work and attitude towards work during a pandemic. There is a need for training of infection control and PPE and minimizing the fear and psychological impact on OHCW should be the priority in any preparedness and planning for combating COVID‐19.

## DISCLOSURE

*Ethical approval*: The research was approved by the ethics committee at HTEC Institute of Medical, Taxila (Reference no. F.23‐11C/2020/ERB/HITEC‐IMS). All procedures performed in this study were in accordance with the institutional and/or national research committee and with the 1964 Helsinki declaration and its later amendments or comparable ethical standards. *Informed consent:* Informed written consent was obtained from all the participants included in the study. *Registry and the registration no. of the study:* N/A. *Animal studies:* N/A. *Conflict of interest:* The authors declare no conflict of interests.

## AUTHOR CONTRIBUTIONS

FAC and BA conceived the idea, performed data analyses and revised the final manuscript; PA and MDK wrote the first draft of the manuscript and revised the final manuscript; DQB and SQK collected the data and provided feedback in the final manuscript.
